# The impact of virtual reality on student engagement in the classroom–a critical review of the literature

**DOI:** 10.3389/fpsyg.2024.1360574

**Published:** 2024-04-10

**Authors:** Xiao Ping Lin, Bin Bin Li, Zhen Ning Yao, Zhi Yang, Minshu Zhang

**Affiliations:** ^1^Faculty of Education, Silpakorn University, Nakhon Pathom, Thailand; ^2^Melbourne Graduate School of Education, The University of Melbourne, Melbourne, VIC, Australia; ^3^Graduate Department, Xi’an Physical Education University, Xi’an, China; ^4^College of Commerce and Tourism, Hunan Vocational College for Nationalities, Yueyang, China; ^5^Graduate Department, Sehan University, Yeongam County, Republic of Korea

**Keywords:** virtual reality technology, cognitive engagement, affective engagement, behavioral engagement, learning outcomes

## Abstract

**Objective:**

The purpose of this review is to identify the impact of virtual reality (VR) technology on student engagement, specifically cognitive engagement, behavioral engagement, and affective engagement.

**Methods:**

A comprehensive search of databases such as Google, Scopus, and Elsevier was conducted to identify English-language articles related to VR and classroom engagement for the period from 2014 to 2023. After systematic screening, 33 articles were finally reviewed.

**Results:**

The use of VR in the classroom is expected to improve student engagement and learning outcomes, and is particularly effective for students with learning disabilities. However, introducing VR into middle school education poses several challenges, including difficulties in the education system to keep up with VR developments, increased demands on students’ digital literacy, and insufficient proficiency of teachers in using VR.

**Conclusion:**

To effectively utilize VR to increase student engagement, we advocate for educational policymakers to provide training and technical support to teachers to ensure that they can fully master and integrate VR to increase student engagement and instructional effectiveness.

## Introduction

In recent years, virtual reality (VR) has emerged as a transformative technology in education, providing new avenues for immersive and interactive learning experiences (Pottle, 2019). At its core, VR offers a departure from the tangible, allowing users to delve into an environment transcending conventional reality (Brooks, 1999; Jeong et al., 2019). VR’s essence is captured in three pillars: presence, interactivity, and immersion (Lee et al., 2017). Presence grants users access to previously unreachable 3D landscapes, facilitating a unique, experiential insight (Poux et al., 2020). Interactivity kindles user curiosity, enabling dynamic engagements within the virtual milieu (Steuer et al. 1995; Huvila, 2013; Song et al., 2023). Immersion pushes the boundaries of conventional experiences, reviving or manifesting phenomena outside the realm of everyday life (Sanchez-Vives and Slater, 2005; Poux et al., 2020).

The introduction of VR in education might increase student engagement, which is closely related to the cognitive, behavioral, and affective dimensions of the engagement model (Wang and Degol, 2014). Cognitive engagement underscores the depth of students’ attention, comprehension, and retention (Wang and Degol, 2014). Behavioral engagement is observable, characterized by consistent attendance and active classroom participation (Wang and Degol, 2014). Affective engagement delves into the emotional realm, encompassing motivation, passion, and learning efficacy (Wang and Degol, 2014).

Existing literature emphasizes the importance of virtual reality technology in promoting full student engagement in cognitive, behavioral, and affective dimensions, and states that the application of virtual reality technology in education has become a trend (Mystakidis et al., 2021). Recent literature indicates a growing adoption of virtual reality in higher education, with adoption rates in UK universities potentially reaching as high as 96% and several institutions in the United States being at the forefront of its implementation (United Kingdom Authority, 2019; Agbo et al., 2021). Harvard University has established dedicated VR laboratories, demonstrating its commitment to educational innovation and advancement through VR (Reid, 1987; Leidner and Jarvenpaa, 1995). This literature shows that the widespread use of VR in education has attracted the attention of a growing number of researchers and educators, with a particular interest in the impact of VR in the classroom in terms of students’ cognitive, behavioral, and affective engagement.

It is worth noting that although existing literature begins to discuss the impact of VR on student engagement, there are still shortcomings in determining the impact of VR on various dimensions of student engagement, which may limit our overall understanding of the topic. Therefore, further discussion is needed to more specifically identify the impact of VR on the various dimensions of student engagement to gain a more comprehensive and concrete understanding. To accomplish this, this review is guided by the following three questions: (1) What are the positive impacts of VR in education? (2) What are the challenges of VR in education? (3) What interventions can address these challenges? With this in mind, the article will first discuss the positive impact of VR on students’ cognitive, behavioral, and affective engagement to help readers understand its potential in education. It will then discuss the challenges facing VR to make constructive recommendations to address the problems in education.

## Method

### Searching strategy

In our methods, we used critical review. According to Grant and Booth (2009) “an effective critical review presents, analyses and synthesizes material from diverse sources”(p.93). Critical perspectives were used to assess the potential of VR in reforming educational practices and improving teaching and learning outcomes. The purpose of this article was to collect literature on the impact of VR on student engagement. Therefore, this article summarizes the previous studies as follows. First, information was obtained from Google, Scopus, and Elsevier databases: “virtual reality,” “cognitive engagement,” “affective engagement,” “behavioral engagement” and “learning outcomes.” The search was limited to articles published between January 2014 and December 2023 in English. The first search used all combinations of the above keywords and, after an initial review, produced 97 potentially relevant articles (Google: 92, Scopus: 3, Elsevier: 2).

In the second phase, secondary terms such as “affect,” “challenge,” and “education” were added, reducing the number of studies to 63 (Google:60, Scopus:1, Elsevier:2). Of these, 34 did not meet the criteria and were excluded. They were excluded because their target audience was teachers and did not discuss the impact of VR on student engagement from the student’s perspective. In the final stage, another 53 articles were excluded because they were repetitive and their purpose was to discuss either technology or engagement, or both. Finally, their full texts were reviewed to determine if their work fits the focus of this article 20 articles (Google: 17, Scopus: 1, Elsevier: 2) qualified for final review, covered a sample on the impact of VR on student engagement, and were included in the analysis.

### Inclusion and exclusion criteria

**Figure 1:**
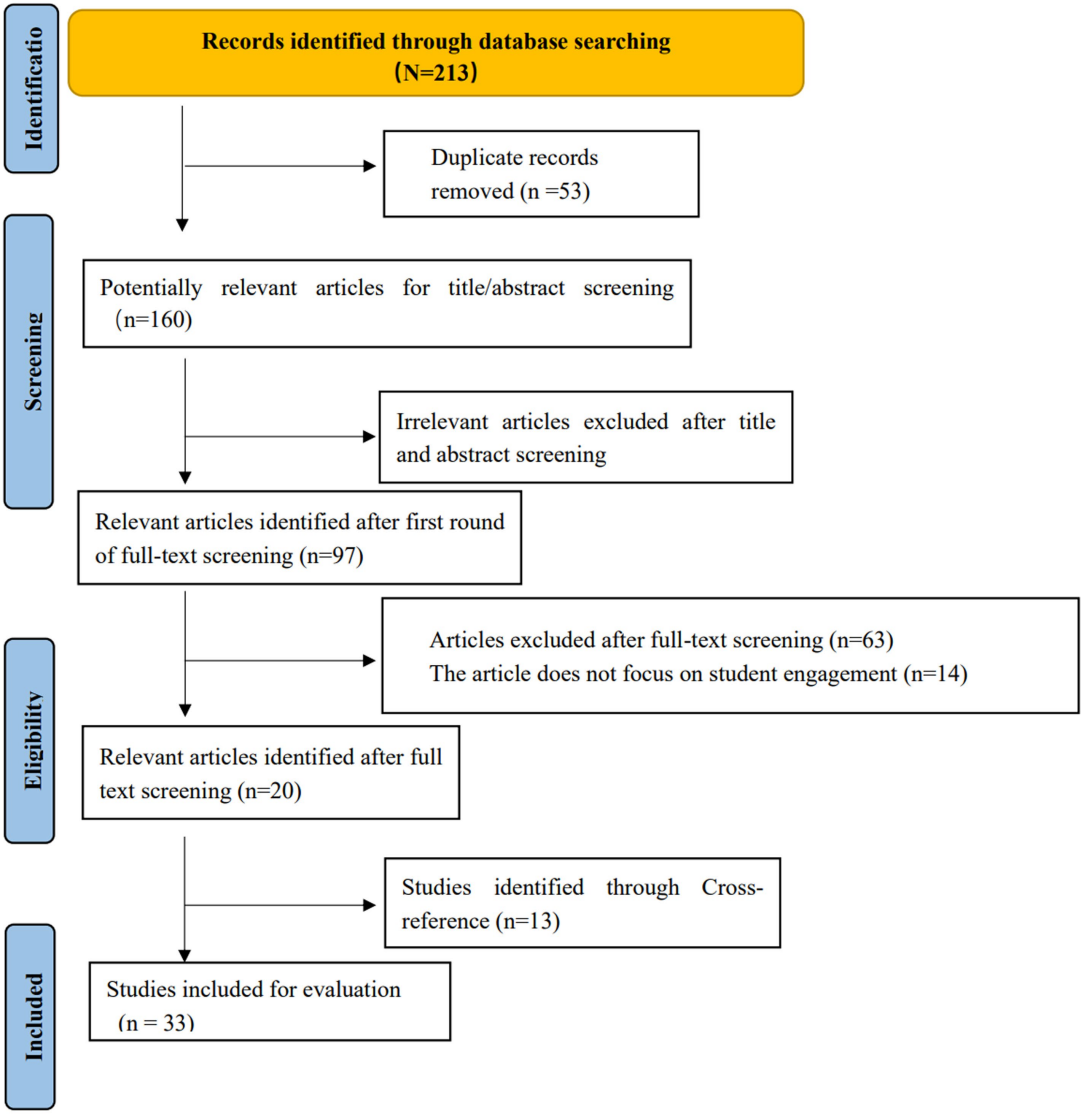
PRISMA flow diagram for article selection.

To ensure the quality of the literature, we selected only peer-reviewed journal articles published in English in the last decade. The main purpose of this article was to review the impact of VR on student engagement. Therefore, we selected only review articles on the impact of VR on student engagement in educational settings. Articles that were not written in English did not discuss the impact on engagement from a student perspective, and were published beyond the previously established time and language were excluded. In addition, a selection of articles was identified and assessed by manually searching the references of articles related to the topic, of which 13 met the eligibility criteria. Therefore, 13 additional articles were added to the 20 identified. In total, 33 articles that met these eligibility criteria were included and reviewed here. Full-text versions of the articles were obtained, with each article being reviewed and confirmed as appropriate by the authors. Finally, to maximize transparency and traceability, we list the rationale and relevant evidence for all articles included (see Table 1). The process of article selection followed the Preferred Reporting of Items for Systematic Reviews and Meta-Analyses (PRISMA) Statement (Moher et al., 2009; see Figure 1). Figure 1 illustrates the process of article selection.

**Table 1:** Publications reviewed in full text with reasons for inclusion or exclusion.

First author	Title	Year	Reason for inclusion
Alfalah	VR in education.	2018	Introducing VR Increased student behavioral engagement.
Allcoat	Learning in VR.	2018	Introduction to learning in VR: effects on cognition, affective, and engagement.
Abich IV	Effectiveness of VR-based training.	2018	The benefits of VR for students are explored.
Cheng	VR in science education.	2015	A systematic review of the use of VR in science education.
Dhimolea	Benefits of VR for learners.	2022	VR is presented as beneficial to learning and increasing learner engagement and learning motivation.
Freina	The state of VR in education.	2015	Presents the advantages and disadvantages of immersive VR in education.
Fransson	The challenge of VR.	2020	Analysis of the challenges of using head-mounted virtual reality in K-12 schools.
Greenwald	The impact of VR on student engagement.	2018	Compares the impact of VR and traditional learning styles on student engagement.
Islam	The challenge of VR.	2015	Point out the VR learning challenges that students face.
Jensen	VR in education.	2018	Critically analyze the use of virtual reality head-mounted displays in education and training.
Lee	VR in education.	2017	Introduces the features of immersive VR as well as its advantages and disadvantages.
Maples-Keller	VR improves student affective engagement.	2017	Introduction to the use of VR to improve students’ mental health and thus their affective engagement.
Misak	VR and meta-cognition.	2018	Introduction to VR improves students’ meta-cognition.
Makransky	VR and Learning.	2019	Describe how adding immersive VR to the classroom will increase student motivation.
Makransky	VR improves affective engagement.	2021	Impact of immersive VR learning on student affective engagement.
Mystakidis	VR-based learning.	2021	An introduction to the benefits of VR-based learning for distance students.
Necci	VR effect.	2015	Introduction to the effects produced by VR.
Pellas	VR learning.	2016	Analyze the theoretical underpinnings and decision-making process of VR’s construction of a sociocultural learning framework.
Papanastasiou	VR implications.	2019	Explores the impact of VR on K-12 students’ 21st century skills.
Pirker	VR education.	2021	Analysis of the potential of VR education.
Reddy	VR advantage.	2020	Introducing VR can improve digital literacy for middle school students.
Radianti	Immersive VR in schools.	2020	A systematic review of immersive VR in schools.
Rospigliosi	VR learning experience.	2022	Introducing VR as a new learning experience for students.
Rojas-Sánchez	VR and education.	2023	Introduction to the use of VR for teaching and learning, VR learning environments, and the use of VR in different areas of knowledge.
Rzanova	VR enhances behavioral engagement.	2023	An introduction to the impact on student behavioral competencies.
Sahlberg	VR implications.	2016	Introduction to the impact of VR technology on schooling.
Schutte	VR enhances affective engagement.	2017	Analysis of improving student empathy through VR to enhance affective engagement.
Sun	VR improves engagement.	2020	Introduction to the effectiveness of VR in increasing engagement among Chinese middle school students.
Som	Advantages of VR in education.	2021	Immersive VR enhances creative learning methods.
Tsivitanidou	VR improves cognitive engagement.	2021	Introduction to the interactive effects of immersive VR in exploring the relationship between students’ cognitive and conceptual gains and attitudinal profiles of engagement.
Wang	VR improves cognitive engagement.	2014	Introducing VR prompts students to stay cognitive engaged and enhances knowledge and research needs for student engagement.
Yuan	VR for learning.	2021	Introduction of VR helps students’ language knowledge and thus enhances cognitive engagement.
Zhong	VR educational leadership.	2017	Analyze VR leadership in the context of K-12 education.

## Result

The review found that the number of publications increased each year from 2014 to 2023, indicating the continued interest of researchers in exploring the impact of VR on student engagement. When reviewing the impact of VR on student engagement, Wang and Degol’s (2014) article had the most citations at 450, suggesting that the article had a strong impact in the area of student use of VR in the classroom. The majority of articles had only 10 or fewer citations, which may have indicated that these articles were relatively new or had less impact in the field. It was worth noting that more recently published articles, such as Rzanova et al. (2023), did not have enough time to accumulate citations, so their impact on the field may not have been fully reflected in current citations.

To summarize, the differences in the number of citations for these articles highlighted their different levels of influence in the area of VR’s impact on student engagement. However, there were some limitations to the review methods. For example, some articles might not have fully reflected their impact on the field in the current citations due to their short time frames, which might have resulted in less comprehensive findings. Furthermore, the literature included was small, and in the future consideration would be given to expanding the search of literature and databases, such as PubMed and Web of Science databases, as well as expanding the search with keywords, such as “students’ attitudes toward VR.” In addition, the inclusion and exclusion criteria might have limited the generalizability of the results of the review, and therefore more caution was needed when generalizing the results of the review.

## The positive impact of VR on education

This section will discuss the impact of VR on students’ cognitive, behavioral, and affective engagement participation. It is important in the field of education. Radianti et al. (2020) noted that student engagement in educational settings was critical to learning outcomes and classroom climate. Yuan and Wang (2021) further noted that the combined effects of cognitive, behavioral, and affective engagement could directly impact student learning outcomes and classroom contextual experiences. Therefore, a deeper understanding of the impact of VR on these three dimensions of engagement can provide valuable insights into educational practices and help educators better optimize classroom environments and teaching methods.

First, Papanastasiou et al. (2019) noted that VR immersive learning experiences promoted students’ cognitive engagement and aided in understanding complex and abstract knowledge. That is, through immersive learning, students can understand and remember what they have learned in greater depth and increase cognitive engagement. Pellas (2016) also found that VR encouraged students to learn through self-directed inquiry and move away from traditional teacher-centered instruction. Pellas (2016) further explained that, through VR scenario reenactments and simulations, students could engage in real-world unavailable learning experiences such as exploring historical sites and visiting distant planets. This means that such learning experiences enable students to explore knowledge in deeper and more varied ways, thus increasing cognitive engagement. Similarly, Maples-Keller et al. (2017) showed that VR was beneficial in engaging different types of students in learning, particularly for at-risk students, including those with learning difficulties, anxiety disorders, and other mental illnesses. VR provided personalized and adaptive learning environments that helped students improve cognitive engagement and achievement (Maples-Keller et al., 2017). In summary, VR facilitates understanding of complex knowledge and promotes cognitive engagement for different types of students through immersive learning experiences and self-directed inquiry learning.

Secondly, Pirker and Dengel (2021) demonstrated that VR could promote student behavioral engagement. They discussed the potential of immersive VR in education through an in-depth analysis of 64 articles. They showed that “learning tasks in 3-D VLEs can foster intrinsic motivation for and engagement with the learning content” (p.77). Sun and Peng (2020) also suggested that by combining classical educational concepts with VR, such as Confucianism’s promotion of teaching for fun, students were better able to engage in learning activities. For example, Rzanova et al. (2023) found that the use of VR in the teaching of poetry to create the scenarios depicted in the verses enabled students to actively participate in classroom activities. Similarly, Freina and Ott (2015) also found that by simulating real school escape scenarios in VR, students could take on different roles to perform escape drills, and this sense of behavioral engagement can help students better master escape techniques and enhance safety awareness. These articles seem to echo that VR helps to enhance student behavioral engagement.

It is worth noting that there is debate about whether VR has a positive impact on student behavioral engagement. Proponents noted that students’ hands-on experience and exploration in virtual environments stimulated interest and behavioral engagement (Wong et al., 2010; Allcoat and Von Mühlenen, 2018). This view suggests that VR provides an immersive learning experience that enhances students’ motivation and promotes deeper engagement in classroom activities. However, contrary findings exist, suggesting that the use of VR may have some negative effects. For example, students might have become addicted to the virtual world and neglected their real-life tasks and responsibilities, thus affecting their behavior in the classroom (Cheng et al., 2015; Greenwald et al., 2018; Makransky et al., 2019). In addition, some other scholars noted that there might have been a gap between learning experiences in virtual environments and real-world learning experiences, which might have affected students’ ability to acquire and apply knowledge (Makransky and Petersen, 2021). These conflicting results remind us that these complexities and diversities need to be taken into account when evaluating the role of VR technology in improving student engagement in the classroom.

Finally, scholars such as Wu et al. (2013), Schutte and Stilinović (2017), and Yuen et al. (2011) found that VR helped to promote student affective engagement. For example, Schutte and Stilinović (2017) found that contexts provided by VR for children with emotional impairments or disabilities taught them skills in communicating with people and managing their emotions, thus fostering empathy. This implies that VR may stimulate affective engagement. Wu et al. (2013) and Yuen et al. (2011) also found that VR provided opportunities for affective interaction, enabling students to interact with characters in the virtual environment. In language learning, for example, practicing through conversations with virtual characters could help students improve their oral expression (Dhimolea et al., 2022). This means that affective interactions may increase students’ affective engagement with the learning content. Similarly, Misak (2018) noted that VR allowed students to role-play in virtual literature and experience the affective portrayed in the story. In other words, affective experiences may deepen students’ understanding of literary works and increase affective engagement. This literature seems to reflect that VR can promote student affective engagement.

In general, VR positively impacts students’ cognitive, behavioral, and affective engagement. In terms of cognitive engagement, VR can facilitate students’ cognitive engagement with learning materials and better understanding of abstract and complex knowledge by creating immersive situations. In terms of behavioral engagement, VR stimulates active student engagement and action through interactive learning. Although there is debate about whether VR has a positive impact on student behavioral engagement, literature has demonstrated the positive impact of VR on student behavioral engagement. In terms of affective engagement, VR promotes students’ emotional engagement by triggering affective resonance through affective experience and affective interaction. This full engagement helps students improve their learning and develop empathy.

The following section discusses the challenges faced when introducing VR in education. Through understanding these challenges, we can better understand the problems in the education system and make some constructive suggestions to help address them.

## The challenge of VR in education

Despite the positive impact of VR on students’ cognitive, behavioral, and affective engagement, there are still two challenges to introducing VR into middle education, namely the difficulty of the educational system in keeping up with VR developments and the lack of teacher proficiency in VR use (Islam et al., 2015; Zhong, 2017; Abich et al., 2021). For example, Islam et al. (2015) observed that the pace of technological advancement, including VR, outpaced the ability of the education system to adapt. This phenomenon is due to the slow reform of the education system, which takes time for the acceptance and adoption of emerging technologies (Islam et al., 2015). To this end, the education sector may take longer to standardize the syllabus, resulting in students not having immediate access to VR (Zhong, 2017). In other words, students may not have the opportunity to experience VR in the classroom until the education department completes the standardization process. Sahlberg (2016) further stated that while reform and standardization in the education sector took time, once VR and the education system evolved in tandem, students benefited from an education that matched the VR of the day.

Other scholars observed that VR education faced several challenges in developing digital literacy in students (Aviram and Eshet-Alkalai, 2006; Sahlberg, 2016). According to Reddy et al. (2020), “digital literacy is a set of skills required by 21st Century individuals to use digital tools to support the achievement of goals in their life situations” (p. 66). Digital literacy encompasses the assessment of digital technologies, critical thinking, and the ability to create and express oneself digitally (Reddy et al., 2020). For example, Tsivitanidou et al. (2021) and Necci et al. (2015) emphasized the need for students to identify the differences between the results of simulation experiments and real experiments and to assess the reliability and accuracy of simulation experiments. In other words, students need to judge the plausibility of the results of simulation experiments and interpret and evaluate those results in real-world situations.

Similarly, Farmer and Farmer (2023) found that digital literacy required students to master VR painting and sculpting tools to create art. This involved learning to select appropriate colors and textures and creating three-dimensional effects with VR tools (Skulmowski et al., 2021). Meanwhile, Andone et al. (2018) further noted that students also needed to learn to share and present their work to others in virtual reality. This observation seems to reflect the high demand for students’ creativity, technical skills, and expressive abilities when introducing VR into education. In sum, while the development of VR education benefits students’ learning in conjunction with VR, there are challenges to students’ digital literacy and the technological adaptability of the education system.

In addition, teachers’ lack of proficiency in the use of VR is another major challenge in introducing VR into middle education. For example, Abich et al. (2021) found that teachers might lack proficiency in the operation and application of VR, which might result in teachers not being able to fully utilize VR to supplement instruction. Jensen and Konradsen (2018) claimed that “for HMDs to become a relevant tool for instructors they must have the ability to produce and edit their content” (p.1525). This means that teachers need to spend time familiarizing themselves with HMDs and related software to create, edit, and customize content to meet their specific instructional needs. Similarly, Fransson et al. (2020) discussed the challenges of teachers operating VR equipment and software. They interviewed 28 teachers to understand teachers’ challenges with implementing helmet display VR in educational settings. Fransson et al. (2020) indicated that there might be a technological threshold and learning curve for teachers in controlling and operating VR devices, which might affect the effective use of VR for teaching and learning.

While teachers may lack familiarity with VR, there are solutions to this challenge. For example, Alfalah (2018) noted that proper training and support could help teachers make the most of VR to supplement instruction. That is, teacher training can provide teachers with the technical knowledge and operational skills they need to familiarize themselves with how VR equipment and software work. To this end, Alfalah (2018) found the impact of providing teachers with VR training in schools. They used a quantitative approach by distributing a questionnaire online to 30 IT teachers. Alfalah (2018) indicated that “technology training may be maximized for the integration of VR technology” (P.2634). This finding seems to reflect that proper teacher training and support can be effective in helping teachers overcome the operational and application of VR technology’s difficulties.

In sum, prior literature has shown that introducing VR into middle school education faces several challenges. First, the rapid development of technology makes the educational system keep up with VR, resulting in a disconnect between the educational curriculum and VR. Second, there may be a lack of proficiency in students’ digital literacy and teachers’ handling and application of VR. However, these challenges are not insurmountable. With proper training and support, teachers can make full use of VR to supplement their teaching and learning to realize the potential of VR in education. It is worth noting that through the literature we have found that in practice, due to the rapid development of technology and the limitations of the educational system, achieving a complete balance may take some time and effort. Therefore, considering how to address the gap between the speed of VR development and the education system to better integrate and apply VR in education makes sense.

## Conclusion

This article describes the impact of VR on student cognitive, behavioral, and affective engagement and the challenges posed by VR education. The literature review finds that using VR in the classroom can positively impact student engagement and learning outcomes. An interesting finding is that VR can be a promising tool for providing education to students with learning disabilities. For example, the previous literature review section describes how for students with learning difficulties, anxiety disorders, and other mental illnesses, VR can provide personalized and adaptive learning environments that can help students improve cognitive engagement and academic performance. And, for children with emotional disorders or disabilities, VR provides contexts that can teach them skills for communicating with others and managing their emotions, thereby developing empathy and stimulating affective engagement.

However, the potential problems with incorporating VR in middle education are the difficulty of the education system in keeping up with VR developments, the higher demands of student digital literacy, and the lack of teacher proficiency in the use of VR. These challenges require educational policymakers to provide training and technical support to teachers to ensure that they can fully master and integrate VR to improve student engagement and teaching effectiveness.

## Author contributions

XL: Writing – original draft, Writing – review & editing. BL: Conceptualization, Writing – original draft, Writing – review & editing. ZNY: Writing – original draft, Writing – review & editing. ZY: Funding acquisition, Supervision, Writing – original draft, Writing – review & editing. MZ: Funding acquisition, Writing – original draft, Writing – review & editing, Supervision.
