# Correction: The impact of virtual reality on student engagement in the classroom–a critical review of the literature

**DOI:** 10.3389/fpsyg.2025.1673859

**Published:** 2025-10-09

**Authors:** Xiao Ping Lin, Bin Bin Li, Zhen Ning Yao, Zhi Yang, Minshu Zhang

**Affiliations:** ^1^Faculty of Education, Silpakorn University, Nakhon Pathom, Thailand; ^2^Melbourne Graduate School of Education, The University of Melbourne, Melbourne, VIC, Australia; ^3^Graduate Department, Xi'an Physical Education University, Xi'an, China; ^4^College of Commerce and Tourism, Hunan Vocational College for Nationalities, Yueyang, China; ^5^Graduate Department, Sehan University, Yeongam County, Republic of Korea

**Keywords:** virtual reality technology, cognitive engagement, affective engagement, behavioral engagement, learning outcomes

An incorrect **Funding** statement was provided. The correct funding statement reads:

The author(s) declare financial support was received for the research and/or publication of this article. This study was supported by the Social Science Achievement Evaluation Committee of Hunan Province, China (Self-Funded General Project, Grant No. XSP2023JYC123). The corresponding author, Minshu Zhang, is the principal investigator of this project. Funding for self-funded general projects typically comes either from the investigator personally or from their affiliated institution.

A correction has been made to the section **Introduction**, page 2, *paragraph 2*, lines 5–11:

“Recent literature indicates a growing adoption of virtual reality in higher education, with adoption rates in UK universities potentially reaching as high as 96% and several institutions in the United States being at the forefront of its implementation (United Kingdom Authority, 2019; Agbo et al., 2021). Harvard University has established dedicated VR laboratories, demonstrating its commitment to educational innovation and advancement through VR (Reid, 1987; Leidner and Jarvenpaa, 1995).”

“Farmer and Farmer 2023” was cited in the article, but its corresponding reference was missing from the reference list. This missing reference has now been added.

“Farmer, L. S., and Farmer, C. J. (2023). “Visual cultural arts as a gateway to digital literacy,” in *Innovative Digital Practices and Globalization in Higher Education*, ed. J. Keengwe (London: IGI Global Scientific Publishing), 16–35. doi: 10.4018/978-1-6684-6339-0.ch002.”

Author Minshu Zhang was erroneously spelled as Mingshu Zhang.

The original version of this article has been updated.

